# The effect of enzymes on release of trace elements in feedstuffs based on in vitro digestion model for monogastric livestock

**DOI:** 10.1186/s40104-018-0289-2

**Published:** 2018-10-15

**Authors:** Xiaonan Yu, Jianan Han, Haiyun Li, Yiwei Zhang, Jie Feng

**Affiliations:** Key Laboratory of Animal Nutrition & Feed Science, College of Animal Science, Zhejiang University, Hangzhou, 310012 People’s Republic of China

**Keywords:** Feedstuffs, Feed enzymes, In vitro model, Release rate, Trace elements

## Abstract

**Background:**

This experiment was conducted to study the effect of different feed enzymes (phytase, xylanase, β-glucanase) on release rate of trace elements (Fe, Cu, Mn and Zn) in 6 commonly used feedstuffs (corn, wheat, barley, soybean meal, wheat bran, wheat middlings) by using an in vitro model, simulating the digestive processes in stomach for 2 h and then in small intestine for 6 h at 39 °C.

**Results:**

Phytase raised (*P* < 0.05) the release rate of Cu and Zn in corn, Cu, Zn and Mn in wheat, Cu in barley, Cu, Zn and Mn in soybean meal, Zn, Fe in wheat bran and Zn, Fe, Mn in wheat middlings. The release rate of various trace elements in feedstuffs was increased after xylanase addition. Compared with the control group, the release rate of soluble Cu in corn, wheat, barley and soybean meal, soluble Zn in corn, wheat and wheat middlings and soluble of Mn in corn, wheat, barley and wheat bran increased (*P* < 0.05) after xylanase treatment. After the treatment of β-glucanase, the release rate of soluble Cu in corn, wheat and wheat bran, soluble Fe in barley, soybean meal and wheat bran and soluble Mn in corn and wheat bran all increased (*P* < 0.05) compared with the control group. In each feedstuff, after corresponding enzyme treatment, the contents of phytic acid, xylan and β-glucan were significantly lower than those of the control group (*P* < 0.05).

**Conclusions:**

Results showed that bound trace elements in feedstuffs can be released by feed enzymes. It may be necessary to take the trace elements in feedstuffs into account in the actual feed preparation including feed enzymes.

## Background

Trace elements are essential nutrients for animal growth. They play critical roles in various biochemical processes and functions and consequently, are generally supplemented as inorganic forms in the livestock diets [1]. The bioavailability of inorganic trace elements such as Cu and Zn is low, about 7%~ 15% and 5%~ 10%, respectively, in monogastric animals, which result in a large amount of trace elements (more than 95%) being excreted in feces and urine [2–4] to eventually reach ground water and soil. After years of manure spreading for agriculture purposes, trace elements may accumulate in soil at toxic levels, the feed and food plants grown on the metal-contaminated soil will also be polluted, thus bring potential threats to animals and people [4].

Feedstuffs contain a certain amount of trace elements, while often be ignored when configured in the actual feed formulation. This may result from the exist of anti-nutritional factors such as phytic acid and non-starch polysaccharides in feedstuffs, which affect the bioavailability of the trace elements [5–7]. Therefore, study on improving the release rate of trace elements in feedstuffs is of great significance on reducing the supplementation of exogenous trace elements in feed and the emission of them in animals’ feces or urine, thus saving feed costs and protecting environment.

Recently enzyme preparations such as phytase and NSP enzymes have been widely applied in feed industry to hydrolyze corresponding anti-nutritional factors and then improve nutrient digestion and absorption efficiency, for instance, the application of phytase could improve the utilization rate of Ca and P [8–11]. NSP enzyme such as xylanase and β-glucanase can increase apparent ileal digestibility of DM, CP, GE, AA and other nutrients [12–15]. According to the anti-nutritional factors’ binding capacity for trace elements in feedstuffs and reported effects of these enzymes, the release of trace elements in feedstuffs could be promoted by these feed additives. However, few studies have been reported on investigating whether these enzymes could release trace elements from feedstuffs while improving the digestion and utilization efficiency of other nutrients. Thus, this research was conducted to study the effect of different feed enzymes (phytase, xylanase, β-glucanase) on release rate of trace elements (Fe, Cu, Mn and Zn) in 6 commonly used feedstuffs (corn, wheat, barley, soybean meal, wheat bran, wheat middlings) using an in vitro model.

## Methods

### Feedstuffs

There were six different commonly used feedstuffs (corn, wheat, barley, soybean meal, wheat bran and wheat middlings) and each with 6 batches (*n* = 6). All 36 feedstuff samples were crushed and segregated by 40 mesh sieve.

### Determination of trace elements in feedstuffs

Determination of Fe, Cu, Mn and Zn content in feedstuffs was done according to the national standard GB/T 13885–2003 [16]. Briefly, measured every element’s standard solution, reagent blank and test sample respectively by atomic absorption spectrometer (Thermo Scientific ICE 3300), read the concentration of the elements in the sample according to the standard curve. Measured 3 times for each sample and average. Contents of Cu, Zn, Fe and Mn of each sample are listed in Table 1.

**Table 1:** The content of Cu, Zn, Fe, Mn and anti-nutritional factors in feedstuffs

Feedstuffs	Batch	Cu, mg/kg	Zn, mg/kg	Fe, mg/kg	Mn, mg/kg	Phytic acid, g/100 g	Xylan, g/100 g	β-glucan, g/100 g
Corn	1	3.06	15.23	67.98	20.32	1.35	6.14	0.13
	2	3.11	14.98	65.65	18.98	1.15	6.20	0.18
	3	3.02	15.65	51.65	19.12	1.45	6.23	0.21
	4	3.09	15.74	69.98	16.98	1.39	6.15	0.10
	5	3.04	16.57	72.92	20.16	1.27	6.27	0.14
	6	3.55	15.22	18.56	19.24	1.37	6.16	0.12
Wheat	1	7.06	37.98	87.65	52.65	1.52	7.89	0.97
	2	7.12	40.23	90.23	49.65	1.37	8.47	0.73
	3	7.03	39.98	88.98	53.65	1.48	7.29	0.68
	4	7.05	38.32	89.98	55.65	1.46	8.72	0.81
	5	7.20	30.41	86.16	48.59	1.33	7.84	0.85
	6	7.31	41.54	95.64	60.43	1.41	7.93	0.82
Barley	1	6.12	32.32	62.12	36.65	1.38	5.47	4.34
	2	5.98	33.32	61.98	34.32	1.74	5.69	4.19
	3	6.06	29.65	59.32	32.65	1.53	6.37	4.24
	4	9.11	31.02	60.98	35.98	1.24	5.83	4.56
	5	5.56	17.85	59.01	39.69	1.62	5.15	4.16
	6	7.38	34.32	62.27	30.00	1.47	5.73	4.35
Soybean meal	1	16.65	64.65	187.65	77.65	2.57	6.20	7.12
	2	17.98	63.98	194.32	80.32	2.48	5.76	6.48
	3	18.65	61.98	188.98	74.98	2.03	5.99	6.82
	4	16.98	64.65	179.98	76.98	1.96	6.14	6.73
	5	22.18	69.55	171.73	85.30	2.16	6.03	7.52
	6	15.49	43.69	122.26	53.22	2.04	5.89	6.21
Wheat bran	1	12.98	75.32	231.32	154.32	3.73	19.84	13.62
	2	13.12	73.65	209.98	159.32	3.10	19.07	14.76
	3	13.06	73.49	213.32	152.32	3.27	20.73	13.79
	4	13.03	74.19	221.46	149.98	4.42	21.56	13.97
	5	13.21	75.76	209.76	160.10	3.85	18.69	14.25
	6	13.64	74.59	242.48	144.18	3.77	20.48	14.00
Wheat middlings	1	13.12	84.98	105.32	106.65	2.41	18.05	20.01
	2	12.98	91.32	99.98	110.32	2.46	18.17	20.37
	3	12.89	82.98	112.32	102.65	2.61	17.84	19.64
	4	13.09	85.65	106.65	111.45	2.32	19.47	21.58
	5	13.77	90.92	115.41	171.93	2.55	17.03	18.23
	6	10.87	68.51	100.49	102.35	2.39	17.92	19.85

### Determination of anti-nutritional factors in feedstuffs

The determination of phytic acid content in feedstuffs was according to the “Standard determination of phytic acid in food” [17]. Xylan and glucan contents in all samples were determined according to the Laboratory Analytical Procedure of “Determination of Structural Carbohydrates and Lignin in Biomass” (Version 2012) from National Renewable Energy Laboratory (NREL) [18]. Contents of anti-nutritional factors of each sample are listed in Table 1.

### Digestion of feedstuffs based on the in vitro model

In vitro digestion was done according to S. Boisen’s method [19, 20], can be divided into two steps including the simulated stomach and the small intestine digestion.

#### Simulated stomach digestion

For each sample, accurately weighed 12 copies of 3.0000 ± 0.0010 g finely crushed material into twelve 100 mL conical flasks. Divided them into 4 different treatment: 1) Phytase group: added 0.3 mL phosphate buffer (0.1 mol/L, pH 6.0) containing 300 μg phytase (5,000 U/g, Sun HY) to the sample; 2) Xylanase group: add 0.3 mL phosphate buffer (0.1 mol/L, pH 6.0) containing 75 μg xylanase (40,000 U/g, Sun HY) to the sample; 3) β-glucanase: add 0.3 mL phosphate buffer (0.1 mol/L, pH 6.0) containing 180 μg β-glucanase (5,000 U/g, Sun HY) to the sample; 4) Control: add 0.3 mL phosphate buffer (0.1 mol/L, pH 6.0) without enzyme to the sample. The amount of enzyme added was based on the product specification which meets the actual production requirement. Each flask was added 25 mL phosphate buffer (0.1 mol/L, pH 6.0) and a magnetic rob and kept magnetic stirring for 1 min, then the pH of the mixture was adjusted to 2.0 by adding HCl (1 mol/L). After 1 mL of pepsin solution containing 25 mg pepsin (3,000 NFU/mg, Ameresco No.0685) and 0.5 mL chloramphenicol ethanol solution added, digested in the water bath (SHJ - A6) at 39 °C for 2 h and stirring slowly and continuously to simulate feed digestion in the stomach.

#### Simulated small intestine digestion

After the process of simulated digestion in the stomach, added 10 mL phosphate buffer (0.2 mol/L, pH 6.8) and then adjusted pH to 6.8 by HCl (1 mol/L) or NaOH (1 mol/L). Finally added a magnetic rob and 1 mL the pancreatin solution containing 50 mg porcine pancreatin (≥4 UPS,Sigma No.P-l750) were added into the mixture. Digested for 6 h in water bath (SHJ - A6) at 39 °C, stirring slowly and constantly to simulate feed digestion in the small intestine.

After digestion, filtrated the digestive production through 0.45 μm filter membrane. Supernatant and the residue were collected separately after filtration.

### Determination of trace elements in the supernatant

Put the supernatant in 250 mL conical flask with plug, added 25 mL mixed acid (nitric acid: perchloric acid = 4:1, volume ratio), soaked overnight, and then kept heating until the digestive solution became colorless and transparent or slightly yellow. After cooling, diluted the digested product with 1% nitric acid solution to 50 mL. Determination of trace elements content in the supernatant was done according to the national standard GB/T 13885–2003 [16] as described above.

### Determination of anti-nutritional factors in feedstuffs after digestion

The contents of phytic acid, xylan and β-glucan in feedstuffs after digestion were determined according to the determination of anti-nutritional factors in feedstuffs as described above.

### Data calculation

The release rate of trace elements in feedstuffs were calculated by the following formula:

Release rate (%) = C_s_ / C_T_ × 100%,

where C_s_ is the soluble trace element content which calculated by measured value in the supernatant and C_T_ is the corresponding total trace element content.

The hydrolysis rate of anti-nutritional factors were calculated by the following formula:

Hydrolysis rate (%) =100% –A_D_ / A_F_ × 100%,

where A_D_ is the and A_F_ is the anti-nutritional factors content after digestion, A_F_ is the corresponding anti-nutritional factors in feedstuffs .

### Statistical analysis

The release rate of trace elements and the content of anti-nutritional factors in the feedstuffs after digestion in the feedstuffs after digestion are presented as mean ± standard deviation (SD). Means between groups were compared by one-way analysis of variance and Scheffe’s *post-hoc* test or non-parameter Kruskal-Wallis test (SPSS software, version 21). *P* < 0.05 was considered significant.

## Results

### The release rate of trace elements in corn

The release rate of trace elements after enzyme treatment in corn are shown in Table 2. The release rate of Cu in the phytase group, the xylanase group and the β-glucanase group was 50.94%, 28.97% and 30.92% higher, respectively than in the control group (*P* < 0.05). The release rate of Zn in phytase and xylanase groups were higher than the control group (*P* < 0.05). Xylanase and β-glucanase raised the release rate of Mn.

**Table 2:** The release rate of trace elements in corn

Trace elements	Control group	Enzyme preparation		
		Phytase	Xylanase	β-glucanase
Cu, %	40.09 ± 11.95^c^	91.03 ± 4.61^a^	69.06 ± 6.88^b^	71.01 ± 8.79^b^
Zn, %	60.09 ± 3.39^b^	90.42 ± 5.96^a^	90.63 ± 8.84^a^	64.78 ± 6.19^b^
Fe, %	43.95 ± 8.19	53.70 ± 2.92	50.58 ± 7.88	55.31 ± 5.15
Mn, %	13.12 ± 3.23^c^	17.67 ± 1.98^c^	30.35 ± 2.53^b^	68.52 ± 4.70^a^

^a,b,c^Means values within a row not sharing a common letter differ significantly (*P* < 0.05)

### The release rate of trace elements in wheat

The release rate of trace elements in wheat treated by enzyme preparations are shown in Table 3. The three enzyme preparations had a significant effect on the release of Cu in wheat (*P* < 0.05) and phytase and xylanase increased the release rate of Zn and Mn (*P* < 0.05).

**Table 3:** The release rate of trace elements in wheat

Trace elements	Control group	Enzyme preparation		
		Phytase	Xylanase	β-glucanase
Cu, %	19.61 ± 1.92^d^	52.99 ± 3.99^b^	64.88 ± 3.32^a^	32.02 ± 4.62^c^
Zn, %	7.48 ± 1.66^b^	30.62 ± 2.59^a^	33.66 ± 4.14^a^	11.38 ± 2.79^b^
Fe, %	10.90 ± 0.49^c^	36.49 ± 3.17^b^	50.42 ± 2.24^a^	11.52 ± 0.41^c^
Mn, %	20.68 ± 4.16^b^	46.35 ± 2.47^a^	43.32 ± 4.38^a^	14.99 ± 3.27^b^

^a,b,c,d^Means values within a row not sharing a common letter differ significantly (*P* < 0.05)

### The release rate of trace elements in barley

As can be seen from Table 4, the release rate of Cu in the phytase group and the xylanase group increased by 11.57% and 11.96% respectively (*P* < 0.05) compared with the control group. Treatment with β-glucanase increased the release rate of Fe (*P* < 0.05) and xylanase promote the release of Mn in barley (*P* < 0.05).

**Table 4:** The release rate of trace elements in barley

Trace elements	Control group	Enzyme preparation		
		Phytase	Xylanase	β-glucanase
Cu, %	25.85 ± 3.84^b^	37.42 ± 7.59^a^	37.81 ± 4.53^a^	30.19 ± 3.31^ab^
Zn, %	11.91 ± 1.86	20.18 ± 2.18	20.71 ± 3.12	18.99 ± 2.68
Fe, %	31.05 ± 1.56^b^	32.45 ± 2.27^ab^	33.14 ± 2.00^ab^	41.38 ± 4.98^a^
Mn, %	42.26 ± 5.22^b^	47.45 ± 2.30^ab^	54.70 ± 6.54^a^	50.00 ± 4.60^ab^

^a,b^Means values within a row not sharing a common letter differ significantly (*P* < 0.05)

### The release rate of trace elements in soybean meal

Table 5 presents the release rate of trace elements in enzyme-treated soybean meal. The release rate of Cu in the phytase group and xylanase group were significantly higher than in the control group (*P* < 0.05), while Fe release rate was higher in the β-glucanase group. Phytase raised the release rate of Zn and Mn (*P* < 0.05).

**Table 5:** The release rate of trace elements in soybean meal

Trace elements	Control group	Enzyme preparation		
		Phytase	Xylanase	β-glucanase
Cu, %	60.60 ± 5.79^c^	85.91 ± 8.25^ab^	91.06 ± 6.05^a^	74.08 ± 7.07^bc^
Zn, %	53.75 ± 7.34^b^	65.00 ± 3.42^a^	60.81 ± 4.49^b^	56.19 ± 4.95^b^
Fe, %	27.37 ± 2.48^b^	32.45 ± 2.27^ab^	31.61 ± 1.61^b^	33.23 ± 4.06^a^
Mn, %	15.57 ± 1.68^b^	21.07 ± 0.94^a^	16.73 ± 1.14^b^	17.34 ± 1.90^b^

^a,b,c^Means values within a row not sharing a common letter differ significantly (*P* < 0.05)

### The release rate of trace elements in wheat bran

As shown in Table 6, all enzyme preparations could significantly increase the release rate of Mn (*P* < 0.05). Compared with the control group, the release rate of Fe in phytase group and β-glucanase group increased by 11.33% and 6.72% respectively (*P* < 0.05), the release rate of Zn in phytase group increased by 48.82% (*P* < 0.05) and the release rate of Cu in β-glucanase group increased by 57.00% (*P* < 0.05).

**Table 6:** The release rate of trace elements in wheat bran

Trace elements	Control group	Enzyme preparation		
		Phytase	Xylanase	β-glucanase
Cu, %	19.10 ± 1.34^b^	56.35 ± 4.13^ab^	46.15 ± 1.75^bc^	67.92 ± 5.54^a^
Zn, %	5.29 ± 0.70^b^	62.29 ± 3.63^a^	33.65 ± 2.33^ab^	12.09 ± 0.83^ab^
Fe, %	11.33 ± 1.51^b^	21.66 ± 2.55^a^	13.19 ± 2.02^b^	18.05 ± 0.54^a^
Mn, %	6.83 ± 1.11^c^	28.74 ± 1.80^a^	13.97 ± 1.57^b^	14.98 ± 1.60^b^

^a,b,c^Means values within a row not sharing a common letter differ significantly (*P* < 0.05)

### The release rate of trace elements in wheat middlings

The release rate in wheat middlings after being treated by enzyme preparations are listed in Table 7. Compared with the control group, xylanase raised the release rate of Zn (*P* < 0.05) and phytase raised the release rate of Zn, Fe and Mn (*P* < 0.05).

**Table 7:** The release rate of trace elements in wheat middlings

Trace elements	Control group	Enzyme preparation		
		Phytase	Xylanase	β-glucanase
Cu, %	33.77 ± 1.44	35.69 ± 4.45	35.73 ± 2.10	38.83 ± 3.36
Zn, %	11.48 ± 1.42^c^	24.11 ± 3.08^a^	16.98 ± 1.67^b^	12.57 ± 0.68^c^
Fe, %	29.66 ± 1.36^b^	34.28 ± 3.09^a^	33.12 ± 2.06^b^	31.73 ± 2.27^b^
Mn, %	20.64 ± 0.92^b^	29.87 ± 1.73^a^	20.80 ± 1.52^b^	19.07 ± 1.60^b^

^a,b,c^Means values within a row not sharing a common letter differ significantly (*P* < 0.05)

### The digestion of anti-nutritional factors in feedstuffs

**Fig. 1:**
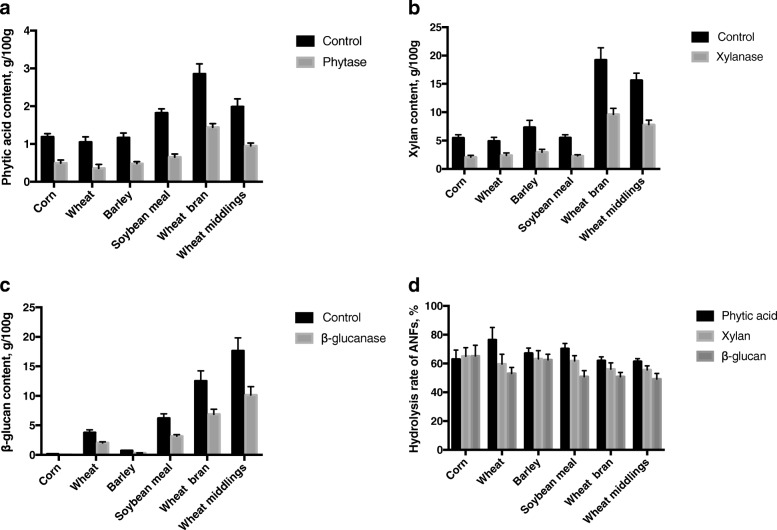
The digestion of anti-nutritional factors in feedstuffs. Values are means ± SD (*n* = 6). **a**, **b** and **c** present the phytic acid, xylan and β-glucan contents in feedstuffs after digestion respectively, **d** presents the hydrolysis rate of anti-nutritional factors after corresponding enzyme treatment. In each feedstuff, after corresponding enzyme treatment, the contents of phytic acid, xylan and β-glucan were significantly lower than those of the control group (*P* < 0.05)

Figure 1 presents the digestion of anti-nutritional factors in feedstuffs. Figure 1a-c present the contents of phytic acid, xylan and β-glucan in feedstuffs after digestion respectively and Fig. 1d presents the hydrolysis rate of anti-nutritional factors after corresponding enzyme treatment. In each feedstuff, after corresponding enzyme treatment, the contents of phytic acid, xylan and β-glucan were significantly lower than those of the control group (*P* < 0.05). In corn, wheat, barley, soybean meal, wheat bran and wheat middlings, the phytic acid content of the phytase group was 58.41%, 66.42%, 58.88%, 64.20%, 49.52% and 52.23% lower than that of the control group, respectively (*P* < 0.05). Compared with the control group, treatment with xylanase reduced the xylan content in corn, wheat, barley, soybean meal, wheat bran and wheat middlings by 61.99%, 51.45%, 59.75%, 58.24%, 49.93% and 50.15%, respectively. The β-glucan content of the β-glucanase group in these feedstuffs decreased by 60.61%, 46.18%, 57.18%, 48.79%, 45.05% and 42.39% respectively compared with the control group. The hydrolysis rate of phytic acid in corn, wheat, barley, soybean meal, wheat bran and wheat middlings is 62.93%, 76.45%, 67.05%, 70.26%, 61.92% and 61.35%, respectively, xylan is 65.07%, 59.60%, 63.21%, 61.80%, 56.07% and 55.50%, respectively, and β-glucan is 65.22%, 53.07%, 62.53%, 50.89%, 50.80% and 49.18%, respectively.

## Discussion

Plant-based feedstuffs contain various kinds of anti-nutritional factors such as phytic acid, xylan, β-glucan, which affect the digestive utilization of nutrients for livestocks. The amount of anti-nutritional factors differs among feedstuffs [5, 6].

Phytic acid, commonly called phytate, also known as inositol hexaphosphate (IP6). It is a strong chelator, which can chelating divalent and trivalent metal ions to form insoluble precipitate, thereby affecting the absorption of these elements. Several reports confirmed that phytase hydrolyze phytate and then release trace elements like Cu and Fe, making them more available to animals. Shelton et al. [21] reported that digestive utilization of Zn, Fe and Mn in pigs have been improved after supplementing diets with phytase. Revy et al. [22] had compared the bioavailability of Zn when piglets were fed with three different diets, basic rations (32 mg/kg Zn) with 20 mg/kg organic zinc, or inorganic zinc or 1,200 U/kg phytase and showed that phytase greatly improved the intestinal bioavailability of Zn and promote effective utilization of Ca, P, Mg, Fe and Cu. In another study [23], it was reported that phytase supplementation of diet increased Zn and Cu bioavailability significantly. Kim et al. [24] found that use of phytase can significantly improve the iron status of piglets and thus reduce adverse performance due to iron deficiency. The results of previous in vivo studies are consistent with the present in vitro one, where phytase greatly raised the release rate of Cu and Zn in corn, Cu, Zn and Mn in wheat, Cu in barley, Cu, Zn and Mn in soybean meal, Zn, Fe in wheat bran, Zn, Fe, Mn in wheat middlings. These results implied that phytic acid can not only inhibit the absorption of Ca and P, but also made it hard for monogastric livestock to absorb trace elements. The results of our research also suggested that phytic acid binds more strongly to Cu and Zn in these feedstuffs. According to the results of the digestion of phytic acid in feedstuffs (Fig. 1a and Fig. 1d) and the results above, it can be speculated that the digestion of phytic acid promotes the release of trace elements in feedstuffs. Therefore, phytic acid may be one of the main anti-nutritional factors which affect the release of trace elements in these feedstuffs.

Xylan is also called the Arab xylan, mainly consisted of Arab sugar and xylose [25]. Xylan constitutes most of cell wall NSP in corn, wheat and barley. Researches showed that the utilization rate of nutrients will be lower when livestocks are fed with excessive feedstuffs containing much xylan such as corn, wheat, barley, bran and so on, which resulted from the anti-nutritional effect of xylan that played the role by improving livestocks’ intestinal chyme viscosity, slowing down the movement speed of chyme in the digestive tract and changing the morphology of digestive tract [26–28]. In addition, as one of the NSP, xylan could easily form complexes with minerals by electrostatic interactions. It will be deprotonated at around neutral pH, and therefore ions like Ca^2+^, Fe^2+^ and Zn^2+^ will interact with the negative charged groups [29]. Xylanase supplementation of diets has been reported to improve the nutrient digestibility such as GE, AA, P, and Ca [30–33]. There is also an in vitro study showing that adding xylanase can release chelated zinc and iron in pearl millet grain [7].Our data showed that the release rate of various trace elements in feedstuffs had been increased after xylanase addition. Compared with the control group, after xylanase treatment, the release rate of Cu in corn, wheat, barley and soybean meal, the release rate of Zn in corn, wheat and wheat middlings and the release rate of Mn in corn, wheat, barley and wheat bran all increased significantly (*P* < 0.05). These findings suggest that xylan is one of the main anti-nutritional factors to bind Cu, Zn and Mn in cereal feed and its byproducts. Besides, xylanase performs better in increasing the release rate of trace elements in corn, wheat and barley. Based on our study on the digestion of xylan in feedstuffs (Fig. 1b and Fig. 1d), xylanase performs well on xylan digestibility in corn, wheat and barley. It could be inferred from the results above that xylan mainly affect the release of Cu, Zn and Mn in corn, wheat and barley.

β-glucan, like xylan, is also a member of NSP. It is present in most cereals and is particularly prevalent in barley and oats. β-glucan consists of a linear chain of glucose monomers linked by β-1-3 and β-1-4 linkages in different ratios [34, 35]. The effects of β-glucan on the absorption of nutrients for livestocks is mainly through the following four aspects: 1) β-glucan encrusting the cell wall and can bind the nutrients in the cell. 2) Its hydrophilicity cause the increase of water layer’s thickness on the intestinal mucosal surface. 3) Due to the presence of β-1-3 linkages, the microfibrils in β-glucan are more tightly packed together, giving them higher solubility than cellulose. Its solubility and considerable length can easily lead to the increase of intestinal viscosity, thus decrease the diffusion rate of enzyme and nutrients and impede well mix of them. 4) It increases chyme viscosity, slow down their movement, thus produce harmful microorganisms and acidic substance which greatly reduce enzymes’ effects [27, 36–38]. In addition, similar to the effect of xylan on electrostatic interactions, it can interact electrostatically with ions like Ca^2+^, Fe^2+^, Zn^2+^ to lower the efficiency of utilization. β-glucanase can crack β-glucan into small molecule and force β-glucan to lose hydrophilicity and thus reduce the viscosity of intestinal contents to improve absorption of nutrition in livestocks. Besides, β-glucanase will release intracellular nutrition by degrading the cell wall. In this study, after the treatment of β-glucanase, the release rate of soluble Cu in corn, wheat and wheat bran, the release rate of Fe in barley, soybean meal and wheat bran, the release rate of Mn in corn and wheat bran all increased significantly compared with the control group (*P* < 0.05). The results showed that β-glucan affect the release of Cu, Fe and Mn in these feedstuffs. Our data also indicated that β-glucanase had better impact on the release of trace elements in corn and wheat bran. After β-glucanase treatment, compared with the control group, the digestion of β-glucan of the β-glucanase group increased in all these feedstuffs (*P* < 0.05). According to the results above, we may infer that β-glucan mainly affect the release of trace elements in corn and wheat bran.

## Conclusion

Anti-nutritional factors like phytate, xylan and β-glucan could inhibit digestion and absorption of trace elements, corresponding enzyme treatment could digest the anti-nutritional factors in conventional feedstuffs and thus release a certain amount of trace elements. Phytase performs better on the release of Cu and Zn in feedstuffs, xylanase has good effect on releasing Cu, Zn and Mn in cereal feed, β-glucanase improve the release rate of Cu, Fe and Mn and it performs better on corn and wheat bran.

## Abbreviations

AA: Amino acid
ANFs: Anti-nutritional factors
CP: Crude protein
DM: Dry matter
GE: Gross energy
NSP: Non-starch polysaccharide
